# Exploratory study on classification of chronic obstructive pulmonary disease combining multi-stage feature fusion and machine learning

**DOI:** 10.1186/s12911-021-01708-2

**Published:** 2021-12-14

**Authors:** Junfeng Peng, Mi Zhou, Kaiqiang Zou, Xiongyong Zhu, Jun Xu, Yi Teng, Feifei Zhang, Guoming Chen

**Affiliations:** 1 School of Computer Science, Guangdong University of Education, Guangzhou, 510006 China; 2 The Third Affiliated Hospital, Sun Yat-sen University, Guangzhou, 510640 China

**Keywords:** Machine learning, Medical decision support system, Real-world data, Chronic obstructive pulmonary disease

## Abstract

**Background:**

Due to the complexity and high heterogeneity of the acute exacerbation of chronic obstructive pulmonary disease (AECOPD), the guidelines (global initiative for chronic obstructive, GOLD) is unable to fully guide the treatment of AECOPD.

**Objectives:**

To provide a rapid treatment in line with the development of the AECOPD after admission. In this paper, we propose a multi-stage feature fusion (MSFF) framework combining machine learning to track the diseases deterioration risk of the AECOPD.

**Methods:**

First, we identify 408 AECOPD patients as the study population. Then, feature segment and fusion methods are applied to generate the phased data set. Finally, human studies are designed to evaluate the performance of the MSFF framework.

**Results:**

The experimental results show that the proposed framework is potential to obtain the full-process tracking of deterioration risk for the AECOPD patients. The proposed MSFF framework achieves a higher overall accuracy average and F1 scores than the four physician groups i.e., IM, Surgery, Emergency, and ICU.

**Conclusions:**

The proposed MSFF model may serve as a useful disease tracking tool to estimate the deterioration risk at each stage, and finally achieve the disease monitoring and management for AECOPD patients.

## Background

Chronic obstructive pulmonary disease (COPD), as a common disease characterized by an irreversible persistent airflow limitation, causes the decline in the quality of life of patients [1, 2]. COPD as a major cause of chronic morbidity and mortality is a global health threat, and will become the third leading cause of death in the world by 2030 [3]. The Acute Exacerbation of COPD (AECPOD) is a key event in the disease course, which causes a sharp decline of lung function and a significant increase in mortality [4, 5]. The increasing frequency of exacerbations and hospitalizations is the daily manifestation of the deterioration of AECOPD [6]. In addition, the prevalence proportion of depressive symptoms is found to be significantly higher among individuals with AECOPD as compared to control [7]. However, timely and reliable risk identification of AECOPD has important clinical significance for prevention and early interventional therapy [8]. In order to identify the deteriorate risk for any given AECOPD patient, physicians often use hypothetical reasoning. From this initial feature set of patients (basic information, past history, current medical history), a physician forms a basic diagnostic set, and then updates the basic diagnostic set based on further clinical data (complications, tests, and examinations) [9]. Thus, the physician who acts like a classifier of sorts reaches final diagnostic set through the cycle of the above process. However, due to the complexity of AECOPD and the lack of consensus, it is a tricky task for clinicians to identify the start of deterioration for AECOPD [10–12]. In addition, the limitations of the human brain and lack of benign statistical and analytical techniques make the identification of acute deterioration events a huge challenge for physicians.

In recent years, Artificial Intelligence (AI) techniques have become potentially powerful tools for the diagnosis and management of diseases, mimicking and even aiding clinical decision-making of human physicians [13–15]. For AI-enabled medical applications, the number of cancer-related publications was the highest, followed by heart diseases and stroke, vision impairment, alzheimers disease, and depression [16]. The intelligent diagnosis of AECOPD requires further research.

To improve the therapeutic effect of the AECOPD, many scholars study the disease based on artificial intelligence. Swaminatha et al. proposed a supervised Gradient Enhanced Random Forest (GERF) model after the collection of the clinical data from AECOPD patients. The prediction accuracy of GERF reaches 88% of that of clinicians [17]. Wang et al. developed a transfer learning algorithm based on balanced probability distribution to predict the risk of exacerbation in patients with AECOPD and the model achieved a good prediction result on the small data sets [18]. Altan et al. used the deep learning method to extract the features of lung sound of AECOPD patients, then the extracted features were utilized to build the model to predict AECOPD. The method achieved 93.67% prediction accuracy [19]. Wang et al. employed a variety of machine learning methods to predict the risk of exacerbation in patients with AECOPD, and the experimental results showed that support vector machine (SVM) performed better than the other models [20]. Ganguly et al. took advantage of the forward feature selection method to select features, and then employed the logistic regression model to achieve the identification of AECOPD. The area under curve (AUC) of the developed model was 0.710 [21]. We can find that the common methods based on artificial intelligence tend to ignore the timeliness of clinical data.

**Fig. 1:**
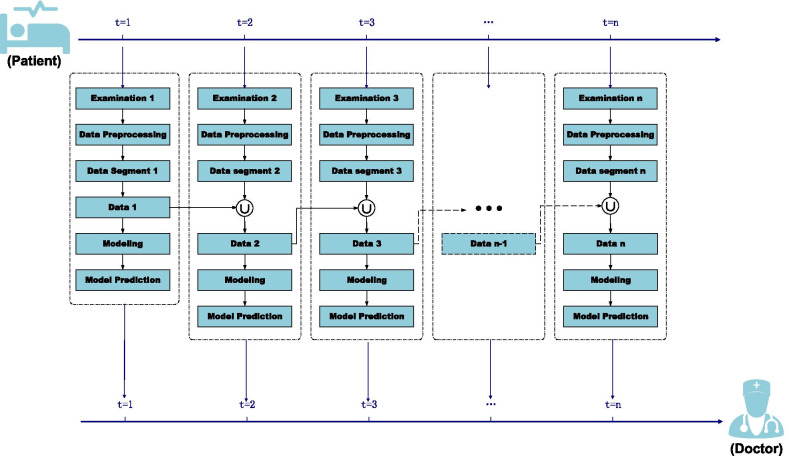
Framework of multi-stage feature fusion

However, the timeliness of clinical data is the key to the real world research (RWD) [22]. The neglect of timeliness of clinical data brings the intelligent diagnosis of AECOPD three challenges at least [23]. First, AECOPD is a highly heterogeneous and complex disease required to be monitored in time. Second, a large amount of treatment data is produced over time. As the treatments progress, a lot of important clinical data sets are gradually produced by time. The mining of the generation time in these clinical data is supportive of clinical decision-making. Third, clinical data is strictly time-sensitive which means that the diagnosis significance of expired data is little. Thus, a model considering the timeliness of clinical data has practical clinical significance to track the diseases deterioration risk. Thus, we propose a multi-stage feature fusion (MSFF) framework to achieve this goal (Fig. 1).

The experimental results show that the proposed framework is potential to obtain the full-process tracking of deterioration risk for the AECOPD patients. The framework can also be extended to the deterioration risk monitoring for the other chronic diseases. The main contributions of this paper are summarized as follows: (1) A method based on machine learning is proposed to track the disease deterioration risk of the AECOPD with the generation time of clinical data. (2) We evaluate the proposed method by real data set from the Third Affiliated Hospital, Sun Yat-sen University.

The remainder of this paper is organized as follows. Section 2 designs the multi-stage feature fusion for deterioration risk trace. In Sect. 3, the evaluation of the proposed framework is carried out. Discussion are provided to the proposed MSFF framework in Sect. 4. We conclude our work in Sect. 5.

## Methods

### Study participants

We conduct the research under the guidance of the Third Affiliated Hospital, Sun Yat-sen University (TAHSYU) Institutional Review Board (IRB), protocol [2019]-02-334-01. 408 AECOPD patients are identified retrospectively by the International Classification of Diseases, Tenth Revision, Clinical Modification (J44.100, J44.101) from the respiratory unit database of TAHSYU, a major Chinese large-scale Medical Center. Data masking techniques are applied to the AECOPD patients before analysis.

The distribution of AECOPD patients is shown in Table 1. The AECOPD inpatients those need the intensive care unit are marked as serious group, while those don’t need as mild group. We can find that the proportion of AECOPD patients with serious group is 46.1%, while the proportion of mild group is 53.9%.

**Table 1:** Basic information of study participants (values are expressed as mean ± standard deviation)

	Mild group	Serious group
Number of cases	220 (53.9%)	188 (46.1%)
Smoking	173 (78.6%)	132 (70.2%)
Age	78.8 ± 9.1	81.5 ± 9.2
Sex (male)	188 (85.5%)	157 (83.5%)
Sex (female)	32 (14.5%)	31 (16.5%)
Number of hospitalization	3.6 ± 2.6	6.5 ± 7.3
Temperate	36.8 ± 0.5	36.7 ± 0.6
Respiratory rate	21.7 ± 3.2	24.7 ± 6.4
Systolic pressure	133.1 ± 19.1	131.6 ± 24.7
Diastolic pressure	75.8 ± 12.3	74.4 ± 13.4
Cor pulmonary	43 (19.55%)	64 (34.04%)
Bronchiectasis	13 (5.91%)	8 (4.26%)
Hypertension	96 (43.64%)	73 (38.83%)
Diabetes	21 (9.55%)	34 (18.09%)
Coronary disease	24 (10.91%)	29 (15.43%)
Chronic kidney diseases	6 (2.73%)	4 (2.13%)
Malignant tumour	18 (8.18%)	16 (8.51%)
Cerebrovascular disease1	9 (4.09%)	8 (4.26%)
Viral hepatitis	3 (1.36%)	2 (1.06%)
Liver cirrhosis	1 (0.45%)	1 (0.53%)

### Feature fusion

We define the data set of AECOPD patients as *P*. A patient in the collection can be defined as $P_i$. We assume that $P_i$ contains *n* clinical features, then $P_i$ can be express as:

1 $$\begin{aligned} p_i = (p_i^1,p_i^2,...,p_i^n), \end{aligned}$$

where $p_i \in P$, $n \in N$. Meanwhile, we assume $p_i^n$ as the clinical feature generated through *k* periods (phases or stages), so that the clinical features of $p_i$ can be divided into *k* parts by periods, and then the clinic data of $p_i$ with multiple time periods can be denoted as:

2 $$\begin{aligned} p_i = ( p_{i\_1}^1, p_{i\_1}^2,..., p_{i\_1}^m, p_{i\_2}^1,...,p_{i\_2}^m,..., p_{i\_k}^1,...,p_{i\_k}^m), \end{aligned}$$

where $p_{i\_k}^n$ represents the clinical data of the patient *i* produced at period phase? *k*. We use $y_{i\_k}$ to indicate the severity level of the AECOPD patient $p_i$ . Then, we can define the phased deterioration risk as:

3 $$\begin{aligned} p_i = (p_{i\_1}^1, ..., p_{i\_1}^n, y_{i\_1}, ,..., p_{i\_k}^1,...,p_{i\_k}^n,y_{i\_n}), \end{aligned}$$

Let $y_{i\_1}=y_{i\_2}=...=y_{i\_n}$. so that we can predict the final deterioration risk based on the phased clinical data. We define $seg_{i\_k}^w$ as (5) to the represent all the clinical features (*w* ) generated at phase *k* of the patient.

4 $$\begin{aligned} seg_{i\_k}^w = (p_{i\_k}^1,p_{i\_k}^2,...,p_{i\_k}^w), \end{aligned}$$

where the number of the collected clinical features at phase *k* can be represented as $w=c(k)$. Thus, we can define the single patient time-based phased accumulating clinical data in the previous *k* stages as $acc_{i\_k}$ shown by Eq. (6).

5 $$\begin{aligned} acc_{i\_k}=(seg_{i\_1}^{c(1)},seg_{i\_2}^{c(2)},...,seg_{i\_k}^{c(k)}). \end{aligned}$$

While all the patients’ time-based phased accumulating clinical data can be expressed by:

6 $$\begin{aligned} D_k= (seg_1,seg_2,...,seg_k), \end{aligned}$$

where $D_k$ and $y_i$ are the k-th input and output of the model, respectively.

While all the patients’ time-based phased accumulating clinical data can be expressed by:

7 $$\begin{aligned} D_k= (D_{k-1},seg_k), \end{aligned}$$

where $D_{k-1}$ indicates the input of stage $k-1$, $seg_k$ denotes the k-th input of stage *k*, respectively.

### Modeling

Data modeling begins after the feature fusion. Assuming that there is a hypothesis *f* on the AEOCPD patient data set *D* through machine learning method. Then, the prediction of the staged deterioration risk for the AECOPD patient $p_i$ is calculated by:

8 $$\begin{aligned} \hat{Y_k}=f(D_k)=f(D_{k-1} , seg_k), \end{aligned}$$

where $\hat{Y_k}$ denotes the deterioration risk of prediction for the AECOPD patient data set. *f* denotes the machine learning model to be solved. $D_k$ represents the AECOPD patient data of stage *k*. ${Y_k}$ depicts the true deterioration risk of the AECOPD patient data set. The difference $\epsilon$ between $\hat{Y_k}$ and ${Y_k}$ can be expressed by:

9 $$\begin{aligned} \epsilon =|\hat{Y_k}-{Y_k}|, \end{aligned}$$

*f* can be simple or complex model. The smaller the difference $\epsilon$ is, the better the model performs. To search the best model *f* from the data set $D_k$, the difference $\epsilon$ should be minimized. In real-world study, considering the complexity of clinical data, it may be more appropriate to build simple models and complex ones together.

### Framework of multi-stage feature fusion

We define the multi-stage feature fusion framework below. In this section, we propose the novel framework by combining the machine learning model and phased accumulating clinical data (Fig. 1). The proposed MSFF framework is presented and discussed in framework 1. The input of the MSFF framework is the phased accumulating clinical data sets shown by Eq. (6). The output is the queue of the phased deterioration risk of the patients. The goal of MSFF framework (Fig. 1) is to obtain the classification results on the phased data set defined by Eq. (6). The framework is as follows. Step 1: set the prediction results empty. Step 2 to step 4: sort the input *D*, segment the input *D* into *K* pieces, and define the cumulative data successively. Step 5 to step 11: for each phased data, construct the machine learning model successively and calculate the prediction results. Step 12: return the phased prediction results.

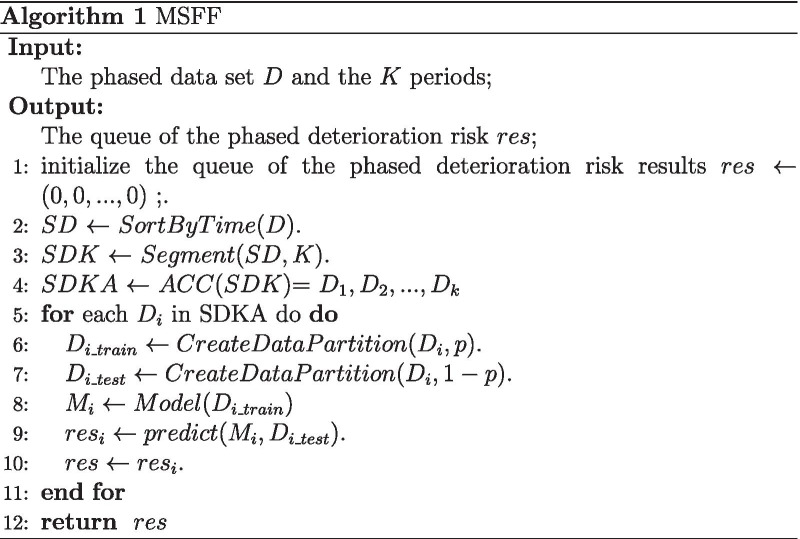



### Human studies

We carry out a study to compare the performance of MSFF framework with that of human physicians on the validation set of data set of $D_4$. We select 16 junior physicians from the department of Internal Medicine (IM), Surgery, Intensive Care Unit (ICU), and Emergency. Each group consists of 4 clinicians (four at the intermediate level and four at the primary level). Each physician in each group reads a random subset of 326 AECOPD patients’ clinical records from the independent training data set and assign a severity rating (serious or mild) to the 82 random validation data set. Then, we evaluate the diagnostic classification performance of each physician group using an $F_1$ score and overall accuracy.

## Results

### Framework initialization

In the real world medical scene, the clinical phase *k* becomes bigger with the development of the treatment. For the convenience of experimental demonstration, we identify 4 segmentations and 40 features as the input of the MSFF framework (shown in Table 2). Then, considering the high accuracy, robustness and fastness of the ensemble of decision trees, random forest model is employed as the model *f* to predict the disease deterioration risk for AECDOP [24, 25]. Finally, we compare our MSFF framework with the clinicians on the phased data set $D_4 = (Seg_1, Seg_2, Seg_3, Seg_4)$. We implemented the MSFF framework using the development platform of R3.5.1. 90% of the whole dataset is randomly separated into the training data set, while the rest of (10%) of the whole dataset is set as independent test dataset. In order to verify the generalization of the model, 10-fold CV is employed. We apply the cross-validated gridsearch approach to optimize the machine learning models and tune the hyperparameters.

**Table 2:** Feature selected in the phased data sets of AECOPD patients

Phase	Data snippet	Description	Feature sets
k = 1	Seg_1	Basics	Gender, age, et al.
k = 2	Seg_2	Comorbidities	Pulmonary heart disease,et al.
k = 3	Seg_3	Inflammations	C-reactive protein, et al.
k = 4	Seg_4	Metabolism	Oxygen saturation, et al.

### Metrics

To evaluate the classifier (Partial least-squares (PLS), Support vector machin (SVM), K-Nearest Neighbor (KNN) and random forest (RF)) of the MSFF model, the overall accuracy and F1-score are used. The F1-measure and the overall accuracy (acc), Sensitive (sn), Specificity (sp), Matthews correlation coefficient (MCC) are the metrics with True Positives (TP), True Negative (TN),False Negatives (FN) and False Positives (FP). The receiver operating characteristic curve (ROC) are also employed to test the MSFF framework. Metrics are defined as follows:

10 $$\begin{aligned} sp= & {} \frac{TN}{TN+FP}, \end{aligned}$$

11 $$\begin{aligned} sn= & {} \frac{TP}{TP+FN}, \end{aligned}$$

12 $$\begin{aligned} acc= & {} \frac{TP+TN}{TP+TN+FP+FN}, \end{aligned}$$

13 $$\begin{aligned} MCC= & {} \frac{TP \times TN - FP \times FN}{\sqrt{(TP+FP)(TP+FN)(TN+FP)(TN+FN)}}. \end{aligned}$$

### Evaluations

**Fig. 2:**
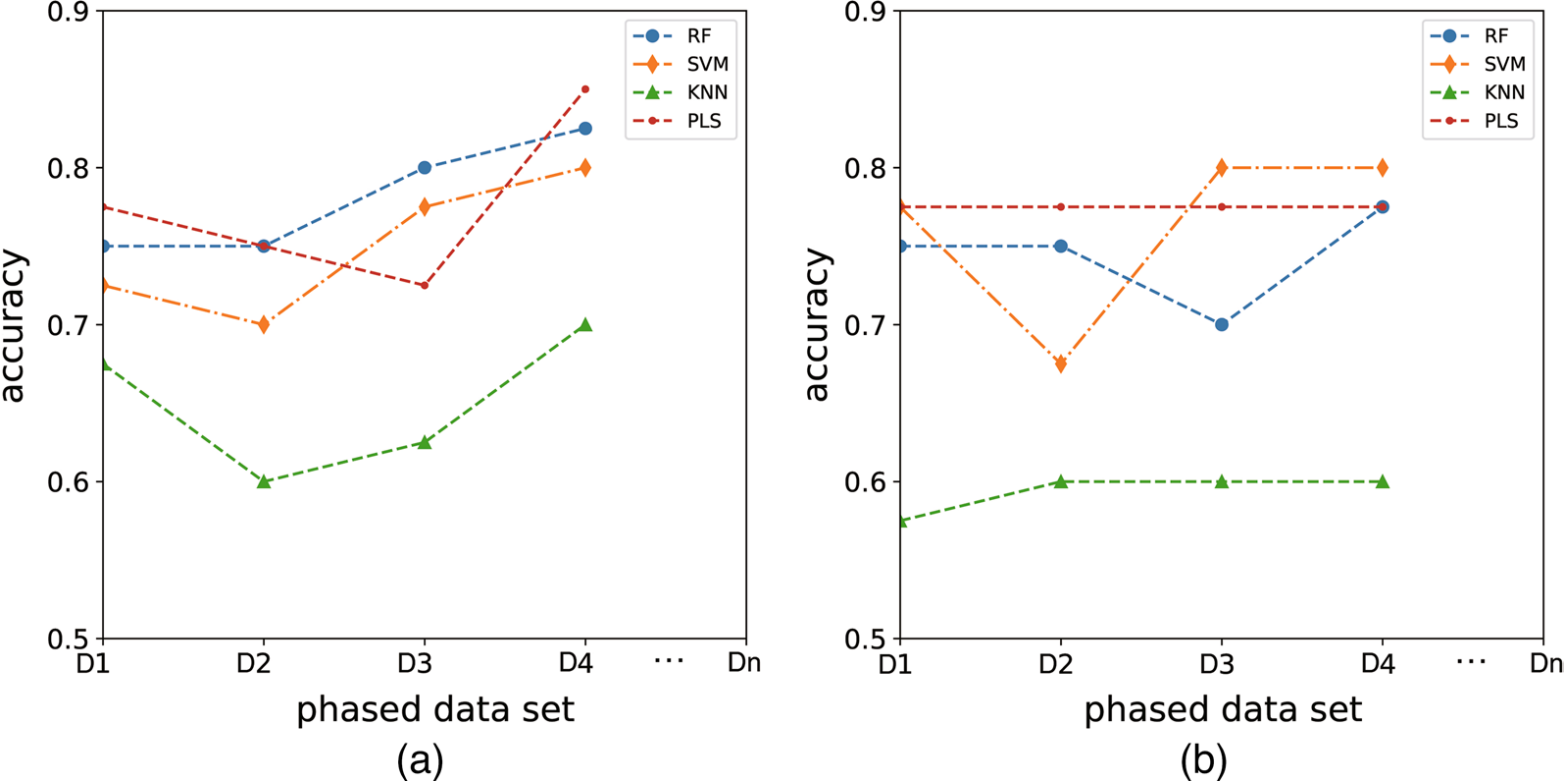
Evaluation of the framework of multi-stage feature fusion with multiple classifiers. **a** Performance evaluation of the MSFF on the stage data D1–D4 generated by cross-validation method. While **b** represents the experimental results by independent test (non-cross-validation method). The x-axis represents the phased data set. The y-axis denotes the prediction accuracy of the proposed framework

To guarantee the robustness of the MSFF framework, both cross-validation and independent test results are provided. Figure 2 and Tables 3, 4 show the evaluation of the classifiers in MSFF framework on the phased data set (D1–D4). Table 3 depicts the performance evaluation of the MSFF on the stage data D1–D4 generated by cross-validation method. The developed RF classifier in the MSFF framework achieves ACC 0.750, 0.750, 0.800, and 0.825 on the four stages, respectively. Similarly, KNN with ACC 0.675, 0600, 0.625 and 0.700. Table 4 denotes the performance evaluation of the MSFF on the stage data D1–D4 generated by the independent test (non-cross-validation method). The developed RF classifier in the MSFF framework achieves acc 0.750, 0.750, 0.700, and 0.775 on the four stages, respectively. Similarly, KNN with ACC 0.575, 0600, 0.600 and 0.600.

**Table 3:** Performance evaluation of the MSFF on the stage data D1–D4 generated by cross-validation method

Phased data set	Model	acc	sn	sp	mcc	Curve
D1	RF	0.750	0.773	0.722	0.495	0.748
	SVM	0.725	0.864	0.556	0.445	0.710
	KNN	0.675	1.000	0.278	0.418	0.639
	PLS	0.775	0.773	0.778	0.548	0.775
D2	RF	0.750	0.773	0.722	0.495	0.748
	SVM	0.700	0.864	0.500	0.395	0.682
	KNN	0.600	0.773	0.389	0.175	0.581
	PLS	0.750	0.818	0.667	0.492	0.742
D3	RF	0.800	0.909	0.667	0.601	0.788
	SVM	0.775	0.818	0.722	0.544	0.770
	KNN	0.625	0.773	0.444	0.231	0.609
	PLS	0.725	0.818	0.611	0.441	0.715
D4	RF	0.825	0.864	0.778	0.646	0.821
	SVM	0.800	0.864	0.722	0.595	0.793
	KNN	0.700	0.909	0.444	0.406	0.677
	PLS	0.850	0.864	0.833	0.697	0.849

D1, D2, D3 and D4 represent the AECOPD patient data of stage 1 to 4. At each stage, there are 408 samples

**Table 4:** Performance evaluation of the MSFF on the stage data D1–D4 generated by independent test (non-cross-validation method)

Phased data set	Model	acc	sn	sp	mcc	Curve
D1	RF	0.750	0.773	0.722	0.495	0.748
	SVM	0.775	0.955	0.556	0.568	0.755
	KNN	0.575	0.773	0.333	0.118	0.553
	PLS	0.775	0.864	0.667	0.545	0.765
D2	RF	0.750	0.773	0.722	0.495	0.748
	SVM	0.675	0.818	0.500	0.338	0.659
	KNN	0.600	0.818	0.333	0.174	0.576
	PLS	0.775	0.864	0.667	0.545	0.765
D3	RF	0.700	0.727	0.667	0.394	0.697
	SVM	0.800	0.864	0.722	0.595	0.793
	KNN	0.600	0.818	0.333	0.174	0.576
	PLS	0.775	0.864	0.667	0.545	0.765
D4	RF	0.775	0.818	0.722	0.544	0.770
	SVM	0.800	0.955	0.611	0.614	0.783
	KNN	0.600	0.818	0.333	0.174	0.576
	PLS	0.775	0.864	0.667	0.545	0.765

D1, D2, D3 and D4 represent the AECOPD patient data of stage 1 to 4. At each stage, there are 408 samples

After a comprehensive comparison of various indicators, it can be found that the developed RF, PLS and SVM classifier in the MSFF framework performs better than KNN classifiers on the four stages, respectively. With the increase of data, the proposed MSFF framework can bring the clinicians the continuous risk observation window. Thus, the mining of the generation time in these clinical data is supportive of clinical decision-making.

On a newly collected data, a contrast experiment between MSFF with RF classifier and clinical physicians on phased data $D_4$. Table 5 shows that the MSFF with RF classifier achieves a higher overall average accuracy and $F_1$ scores than the four junior physician groups i.e., IM, Surgery, Emergency, and ICU. This result shows that the proposed MSFF with RF classifier may potentially support clinic physicians in diagnoses of the deterioration and death risk in patients with AECOPD.

**Table 5:** Diagnostic performance comparison of the junior physicians and MSFF with RF classifier on the new data set

	Overall accuracy	Class	F1 score
Internal medicine	0.667	Serious	0.644
		Mild	0.691
Surgery	0.632	Serious	0.635
		Mild	0.625
Emergency	0.644	Serious	0.617
		Mild	0.663
ICU	0.701	Serious	0.676
		Mild	0.722
MSFF with RF classifier	0.800	Serious	0.765
		Mild	0.826

The feature of the new data is equivalent to the D4

## Discussion

MSFF is conceived to comprehensively integrate the multi-stage clinic data to forecast the exacerbation risks of AECOPD before they occur. Based on a comparison with junior physicians from Internal Medicine (IM), Surgery, Intensive Care Unit (ICU), and Emergency, the proposed MSFF framework obtains superior performance when forecasting the AECOPD exacerbation risk from real world data. There are several issues that require further discussion.

For the convenience of experimental demonstration, the phased data set is defined by the type of clinical indicators, and then the observation stage *K* is determined (Table 2). In general, the number of stages *K* is directly proportional to the severity of the patients’ condition. The number of large stages *K* can achieve the seamless monitoring of the patient’s condition. However, it will consume a lot of computing resources and generate frequent prompts to the clinicians.Thus, it is necessary to determine the balance between computing resource consumption and seamless tracking. In fact, the medical institutions should determine the number of stages *K* according to their own reality.

Further, we need to discuss the model selection problem. First, models used for clinical analysis require over-fitting prevention, noise resistance, and outstanding prognostic performance. The traditional statistical and linear models are highly explanatory. However, the inherent complexity and interactivity of the pathogenic factors of AECOPD make it difficult to use traditional statistical and linear models, e.g., linear causal interpretation. Second, the large number of clinical data generated in stages is a huge challenge for clinicians. Thus, a model with good accuracy and tracking ability is helpful in revealing the degree of influence and correlation with multiple clinical indicators.

The shortcoming of this paper includes lack of tracking data (anxiety and depression) of AECOPD patients after discharge from hospital because of the poor follow-up compliance of the study subjects. Medical imaging was not used during data acquisition as we used the lung function to determine the severity level. In the future, we will establish multicenter contracts to obtain more AECOPD patient data. We hope that the model’s predictive power will be improved by more abundant and reliable real-world training data.

## Conclusions

To achieve the real-time monitoring of acute exacerbation disease such as AECOPD, we digs down the generation time of the clinical features, dynamically divides the clinical data into multiple stages, and utilizes the machine learning methods to perform the deterioration risk warning in stages, eventually achieves the real-time monitoring for the acute exacerbation diseases. The proposed MSFF framework is able to track the phased deterioration risk of AECOPD patients with real world data. Our model achieves a higher classification performance than the four junior physician groups. The data segmentation proposed in this paper conforms to the process of clinical diagnostic reasoning. With the increase of clinical information, the predictive performance of the proposed MSFF framework may be gradually improved. Further work we will investigate the natural language processing technology to dig out potentially valuable information from the electronic medical records, such as the patient’s past history, present medical history, course of illness, discharge summary and other text records [26].

## Data Availability

The data that support the findings of this study are available from the third affiliated hospital, sun yat-sen university but restrictions apply to the availability of these data, which were used under license for the current study, and so are not publicly available. Data are however available from the authors upon reasonable request and with permission of the third affiliated hospital, sun yat-sen university.

## Abbreviations

RWS: Real world search
COPD: Chronic obstructive pulmonary disease
AECOPD: Acute exacerbation of chronic obstructive pulmonary disease
GOLD: Global initiative for chronic obstructive
MSFF: Multi-stage feature fusion
RF: Random forest
SVM: Support vector machine
PLS: Partial least-squares
KNN: K-nearest neighbor
